# Feedback of actionable individual patient prescription data to improve asthma prescribing: pragmatic cluster randomised trial in 233 UK general practices

**DOI:** 10.3399/BJGP.2021.0695

**Published:** 2022-07-12

**Authors:** Sean MacBride-Stewart, Charis Marwick, Margaret Ryan, Bruce Guthrie

**Affiliations:** Pharmacy Services, NHS Greater Glasgow and Clyde, Glasgow.; Department of Population Health and Genomics, University of Dundee, Dundee.; Glasgow School of Business and Society, Glasgow Caledonian University, Glasgow.; Advanced Care Research Centre, University of Edinburgh, Edinburgh.

**Keywords:** asthma, bronchodilator, feedback, general practice, inappropriate prescribing, inhaler

## Abstract

**Background:**

Potentially inappropriate prescribing (PIP) of asthma bronchodilator inhalers is associated with increased morbidity and mortality.

**Aim:**

To evaluate the effectiveness of feedback on the PIP of bronchodilator inhalers.

**Design and setting:**

Pragmatic cluster randomised trial involving 235 of 244 (96.3%) GP practices in one Scottish health board.

**Method:**

Practices were randomly allocated (1:1 ratio) to individualised feedback (including visualised medication histories for each patient and action-oriented messages) on PIP of bronchodilator inhalers from prescription data; feedback reports were sent in July 2015, February 2016, and August 2016. Controls were sent feedback on an unrelated subject. The primary outcome was the change in the mean number of patients per practice with PIP of bronchodilator inhalers from the baseline period (August 2014–July 2015) until the post-feedback period (February 2016–January 2017), identified through a composite of five individual measures using prescription data.

**Results:**

In the analysis of the primary outcome, the mean number of patients with PIP of bronchodilator inhalers fell in the 118 practices that were sent feedback from 21.8 per practice to 17.7 per practice. Numbers fell marginally in the 115 control practices, from 20.5 per practice to 20.2 per practice, with a statistically significant difference between the two groups. There were 3.7 fewer patients per practice with PIP of bronchodilator inhalers in the intervention practices versus the control practices (95% confidence interval = −5.3 to −2.0).

**Conclusion:**

Individualised feedback of PIP of asthma bronchodilators that included background information, visualised medication histories for each patient, and action-oriented messages was effective at reducing the number of patients exposed to excess or unsafe prescribing of bronchodilator inhalers.

## INTRODUCTION

In 2022 the charity Asthma UK reported that 9% of children (1.1 million) and 8% of adults (4.3 million) were receiving treatment for asthma^1^ and, on average, three people die from an asthma attack in the UK every day.^1^ The National Review of Asthma Deaths (NRAD) examined 195 deaths of people with asthma;^2^ two key concerns were the prescribing of large quantities of short-acting β-agonist (SABA) bronchodilators and long-acting β-agonists (LABAs) without inhaled corticosteroids (ICS), both of which have been associated with increased morbidity and mortality.^3^^–^^5^ The prevalence of >12 SABA inhalers per annum in patients with asthma was 10.2% in 2015, with 3.5% being prescribed a SABA only; 0.3% were prescribed LABAs without ICS.^6^ The excessive prescribing of reliever medication, underprescribing of preventer medication, and inappropriate prescribing of LABA bronchodilator inhalers for asthma identified by NRAD could be considered potentially inappropriate prescribing (PIP) that requires review and reflection.^2^

In 2014, access to a national, patient-level prescription database — the Prescribing Information System (PIS)^7^ — was provided to all 14 NHS territorial health boards in Scotland. The PIS holds a record of dispensed medicines linked to patient name, address, and age through the Community Health Index (CHI); however, it is not linked to diagnosis. NHS Greater Glasgow and Clyde health board investigated the use of patient-level prescription data to identify PIP and provide improved prescription data feedback to practices. This study aimed to evaluate the impact of providing practices with feedback on patient-level PIP using nationally available prescription data, and to do so within the resource constraints of the NHS.

## METHOD

### Design

This was a pragmatic cluster randomised controlled trial (RCT) embedded in existing NHS quality-improvement work. The West of Scotland NHS Research Ethics Service assessed the study as service evaluation, and practices were free to respond to feedback as they thought necessary; as such, practice consent was not required. All practices in the health board were randomised to the intervention (feedback on PIP of bronchodilator inhalers) group or to the control group.

### Setting and participants

The trial was conducted in one Scotland NHS health board where all prescribers in the enrolled practices were exposed to the same board-wide and national prescribing policies. The intervention was directed at practices, the cluster unit of randomisation.^8^^,^^9^ Practices were excluded if:

- they had <250 people registered for NHS services (these are all unusual practices in various different ways — for example, they serve people who are homeless);
- <90% of dispensed prescription items for inhaled bronchodilators had a CHI in any month from January 2014 until December 2014; or
- they had been created after 1 January 2015.

**Table:** How this fits in

Feeding back to GPs about their prescribing is a common intervention, but evidence suggests that, alone, it is not very effective at changing behaviour. The authors investigated whether newly available, patient-level prescription data could be used to measure potentially inappropriate prescribing of bronchodilators. This pragmatic study found that patient-level feedback to GPs was effective at reducing the number of patients exposed to excess or unsafe prescribing of bronchodilator inhalers. It would be feasible to implement the giving of such feedback, at scale, where primary care electronic prescribing is in general use.

### Intervention

Details of the intervention design and implementation are given in Supplementary Table S1. In summary, feedback reports were developed, drawing on Ivers *et al*’s s2012 Cochrane systematic review of audit and feedback,^10^ which identified elements associated with more-effective feedback; those elements are outlined in Box 1.

**Box 1.** Implementation of Ivers *et al* ’s^10^ components of effective audit and feedback

**Component**	**Implementation into study**
The source of feedback is a supervisor or colleague	Feedback report sent with signatures from three key lead clinicians in the health board (health board’s clinical director, chair of Primary Care Prescribing Management Group, and lead clinician for Prescribing Services)
Feedback is provided more than once	Feedback sent three times over a 13-month period, with a refreshed up-to-date analysis in each report
Feedback includes both explicit targets and an action plan	Feedback included key messages that supported and encouraged actions expected to be taken by prescribers for the patient with PIP (for example, medication review and/or referral to specialist services) and actions taken in the practice to improve prescribing processes that directly influence PIP (for example, changing the prescription record to increase control of further repeat prescribing)
Baseline performance is low	Ensured PIP present in all practices
Feedback is delivered in both verbal and written formats	Feedback was sent by email to the practice’s secure clinical email address and copied to the practice’s prescribing support team pharmacist (Supplementary Table S1)

*PIP = potentially inappropriate prescribing.*

Each practice was sent a pre-notification letter explaining the feedback programme — in line with a Cochrane systematic review, which found that pre-notification of electronic and postal questionnaires improved response rates^11^ — and then sent a feedback report three times over a 13-month period; practices were followed up to check receipt of each report. The feedback report provided background information and summary data (including how the practice compared with others), along with a visualisation of recent medication history, key measures relevant to the PIP, and an action checklist for each patient in the practice with bronchodilator inhaler PIP detailed analysis (Supplementary Appendix S1).

The control group of practices received similarly structured feedback on an unrelated PIP subject (Supplementary Appendix S2). No specific targets for change in PIP in either the intervention or control practices were set and no incentive payment was made.

Discussion in the practice took place 4 weeks after the first report was received.

### Main outcome measures

The feedback report and outcome measures used PIS data. The PIP examined related to:

- excess SABAs — namely, >12 inhalers per annum with subtherapeutic or no ICS; or
- single-agent LABA inhalers — namely, LABAs with subtherapeutic or no ICS.

As guideline recommendations for asthma management vary according to the patient’s age and the presence of chronic obstructive pulmonary disease (COPD), three SABA indicators and two LABA indicators were defined. To minimise the inclusion of people with COPD, patients aged ≥35 years and prescribed long-acting muscarinic bronchodilators were excluded from these measures; the component measures were:

- aged 5–11 years with excess SABA (>12 SABA inhalers per annum and no inhaler containing single-agent or combination ICS [or average daily exposure of <200 mcg beclometasone or equivalent]);
- aged 12–34 years with excess SABA (>12 SABA inhalers per annum and no inhaler containing single-agent or combination ICS [or average daily exposure of <400 mcg beclometasone or equivalent]);
- aged ≥35 years with excess SABA (>12 SABA inhalers per annum and no long-acting muscarinic antagonist [LAMA] and no inhaler containing single-agent or combination ICS [or average daily exposure of <400 mcg beclometasone or equivalent]);
- aged <35 years with single-agent LABA (≥1 LABA inhaler and no single-agent ICS inhaler [or single-agent ICS inhaler if average daily exposure was <400 mcg beclometasone or equivalent]); and
- aged ≥35 years with single-agent LABA (≥1 LABA inhaler and no LAMA and no single-agent ICS inhaler [or single-agent ICS inhaler if average daily exposure was <400 mcg beclometasone or equivalent]).

The primary outcome was the mean number of patients per practice with any PIP. The five secondary outcomes were the mean number of patients per practice with each of the five component measures of the primary outcome. As individual patients with PIP could differ in each round of feedback, an aggregate measure was used to estimate the intervention effect size.^12^

### Power calculation

A practice-level analysis that measured the change in the mean number of patients with PIP per practice, including all provisionally eligible practices in the health board (*n* = 236), was estimated to have a 90% power to show a 40% reduction in the bronchodilator inhaler PIP outcome (Supplementary Table S2).

### Randomisation

Randomisation was stratified to ensure balance across practice location (the eight localities in the health board) and across low, medium, and high tertiles of baseline PIP (January 2014–December 2014), to give 24 strata. The localities in the health board vary in size and have separate operational responsibility for practice prescribing. Stratifying by the locality was, therefore, necessary in case there had been a prior locality-implemented improvement activity designed to influence PIP; stratifying by PIP at baseline was to achieve balance in baseline performance over the two study arms.

Randomisation was carried out independently by the Tayside Clinical Trials Unit using the SURVEYSELECT procedure of SAS (version 9.3).

### Blinding

The independent statistician was blind to practice details at randomisation. Practices were blinded to the study, and only knew about the report they were sent. The researchers were not blind to the intervention allocation as they distributed the feedback reports to practices; however, they were blinded at the point of analysis. Baseline and post-intervention prescription data for the enrolled practices were extracted by a data analyst in the health board who was blind to practice allocation.

### Process evaluation

Following the intervention period, practices were surveyed to evaluate whether the feedback was read and what actions were taken in response by the practice team (GPs, practice nurses, and practice managers) or members of the NHS-employed prescribing support team (pharmacists and pharmacy technicians). The survey (see Supplementary Table S3 and Supplementary Figure S1) was designed to be answered by anyone likely to have read or actioned the feedback. The survey link was disseminated via email after trial data collection was complete in order to ensure the survey itself did not influence the outcome measure. Survey data was collected using Online Surveys (https://www.onlinesurveys.ac.uk).

### Statistical analysis

The efficacy data were analysed using a practice-level linear regression model to test the statistical significance of the difference of change in the mean number of patients with PIP (see Supplementary Table S4). Adjustments made for baseline performance, locality, and practice deprivation were pre-specified.^13^^,^^14^ Analysis was carried out using the regress procedure with the robust option in Stata (version 13.1).

## RESULTS

Of 244 practices assessed for suitability in May 2015, nine were excluded (two were very small practices, two were newly created, and five had inadequate capture of patient identifiers on prescriptions). There were, therefore, 235 (96.3%) practices randomised in June 2015; 119 intervention practices were sent feedback on the PIP of bronchodilator inhalers and 116 control practices were sent feedback unrelated to that. One intervention practice merged with a control practice during the study period and both were, therefore, lost to follow-up.

Intervention and control practices were similar in terms of: the numbers of registered patients (list size); the mean age, sex, and deprivation status of the registered patients; urban/rural location of practice; and practice locality (health and social care partnership). There was a moderate difference in the proportion of practices that were accredited for training: 36 (30.5%) intervention practices compared with 45 (39.1%) control practices (Table 1).

**Table 1.** Baseline characteristics of practices, July 2015

**Characteristic**	**Control group**	**Intervention group**
**Patients**		
Total, *n* (%)	2357 (100)	2572 (100)
Males with PIP, *n* (%)	1172 (49.7)	1291 (50.2)
Age, males, years, mean (SD)	42.9 (20.0)	43.2 (19.6)
Age, females, years, mean (SD)	48.7 (20.0)	47.7 (19.6)
Living in most deprived 15% of data zones, *n* (%)	941 (39.9)	1038 (40.4)

**Practices**		
Total, *n* (%)	115 (100.0)	118 (100.0)
Mean list size, *n* (SD)	4998 (2527.3)	5173 (2526.1)
Accredited for training, *n* (%)	45 (39.1)	36 (30.5)
**Level of deprivation,^a^ *n* (%)**		
<33% of registered patients living in most deprived data zones	60 (52.2)	62 (52.5)
33%–66% of registered patients living in most deprived data zones	44 (38.3)	42 (35.6)
>66% of registered patients living in most deprived data zones	11 (9.6)	14 (11.9)
**Practice location,^b^ *n* (%)**		
Large urban area	95 (82.6)	99 (83.9)
Other urban area	15 (13.0)	17 (14.4)
Accessible small town	3 (2.6)	2 (1.7)
Accessible rural	2 (1.7)	0 (0.0)
**Locality,^c^ *n* (%)**		
A	9 (7.8)	8 (6.8)
B	8 (7.0)	7 (5.9)
C	20 (17.4)	21 (17.8)
D	24 (20.9)	26 (22.0)
E	23 (20.0)	26 (22.0)
F	9 (7.8)	7 (5.9)
G	13 (11.3)	15 (12.7)
H	9 (7.8)	8 (6.8)

a *Practices with registered patients living in most deprived areas (% of patients with postcode in 15% most deprived data zones).*

b *Large urban area = settlement of >125 000 people; other urban area = settlement of 10 000–125 000 people; accessible small town = settlement of 3000–10 000 people and within 30-minute drive of a settlement of ≥10 000 people; and accessible rural = settlement of <3000 people and within a 30-minute drive of a settlement of ≥10 000 people.*

c *Health and social care partnerships in the health board, each with separate operational responsibility for the prescribing in the practices of their specific area. PIP = potentially inappropriate prescribing. SD = standard deviation.*

**Figure 1.**
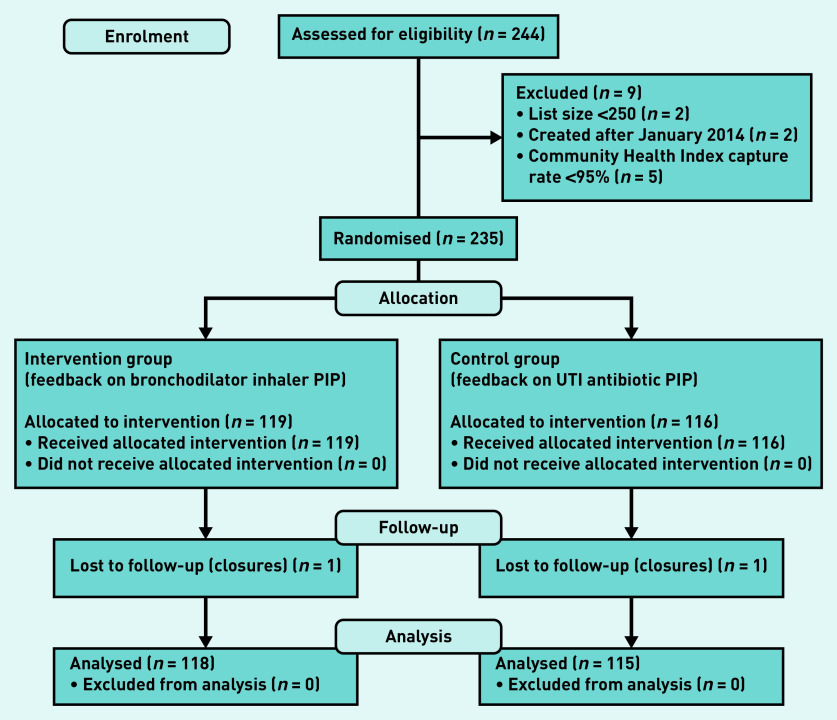
**CONSORT flow diagram of the study. PIP = potentially inappropriate prescribing. UTI = urinary tract infection.**

Data for 118 (99.2%) intervention practices and 115 (99.1%) control practices were analysed (Figure 1).

### Primary outcomes

The mean number of patients with PIP of bronchodilator inhalers decreased from 21.8 to 17.7 in the intervention practices and from 20.5 to 20.2 in control practices. There was a statistically significant difference of 3.7 fewer patients per practice with PIP in intervention practices compared with control practices (95% confidence interval = −5.3 to −2.0) (Table 2).

**Table 2.** Primary outcome: change in the mean number of patients per practice with PIP of bronchodilator inhalers from baseline (August 2014–July 2015) until post-feedback period (February 2016–January 2017)

**Outcome**	**Control group**	**Intervention group**	**Difference^a^ (95% CI)**
Total, *n*	115	118	—

Patients per practice with			
PIP of individual bronchodilator inhaler, mean (SD)			
Baseline	20.5 (14.9)	21.8 (13.6)	−3.7
Post-intervention	20.2 (14.8)	17.7 (11.8)	(−5.3 to −2.0)

a *Mean change in count of patients adjusted using full pre-specified model, including baseline mean number patients with PIP, proportion of patients living in deprivation, and GP practice locality. CI = confidence interval. PIP = potentially inappropriate prescribing. SD = standard deviation.*

### Secondary outcomes

Although the study was not powered to detect changes in the different component measures of the PIP of bronchodilator inhalers, there were statistically significant reductions in intervention practices compared with control practices in four of the five secondary outcomes (Table 3). In the group with the smallest change (children aged 5–11 years with asthma who were prescribed multiple SABA inhalers), there was no difference between intervention and control practices.

**Table 3.** Secondary outcomes: component measures of PIP of individual bronchodilators

**Component measure**	**Stage**	**Control group, mean (SD)**	**Intervention group, mean (SD)**	**Difference^a^ (95% CI)**
Total patients, *n*	*—*	115	118	—

**Patients per practice aged 5–11 years with excess SABA**	Baseline^c^	0.3 (0.6)	0.3 (0.5)	−0.05 (−0.19 to 0.09)
>12 SABA inhalers per annum and no ICS-containing inhalers^b^ (or average daily exposure <200 mcg of beclometasone or equivalent)	Post-intervention^c^	0.3 (0.6)	0.3 (0.5)	*—*

**Patients per practice aged 12–34 years with excess SABA**	Baseline^c^	4.3 (3.5)	4.2 (3.0)	−0.56 (−1.07 to −0.04)
>12 SABA inhalers per annum and no ICS-containing inhalers^b^ (or average daily exposure <400 mcg of beclometasone or equivalent)	Post-intervention^c^	3.9 (3.1)	3.2 (2.6)	*—*

**Patients per practice aged**≥**35 years with excess SABA**	Baseline^c^	9.6 (7.1)	9.7 (6.6)	−1.6 (−2.5 to −0.6)
>12 SABA inhalers per annum and no LAMA and no ICS-containing inhalers^b^ (or average daily exposure <400 mcg of beclometasone or equivalent)	Post-intervention^c^	9.5 (7.2)	8.1 (6.1)	*—*

**Patients per practice aged** <**35 years with single-agent LABA**	Baseline ^c^	1.7 (2.4)	2.0 (2.6)	−0.31 (−0.61 to −0.01)
≥1 LABA inhaler and no single-agent ICS inhalers (or single-agent ICS inhalers where average daily exposure <400 mcg of beclometasone or equivalent)	Post-intervention^c^	1.3 (1.9)	1.1 (1.4)	*—*

**Patients per practice aged 35 years with single-agent LABA**	Baseline^c^	5.1 (5.8)	6.1 (5.8)	−1.1 (−1.9 to −0.2)
≥1 LABA inhaler and no LAMA and no single-agent ICS inhalers (or single-agent ICS inhalers where average daily exposure <400 mcg of beclometasone or equivalent)	Post-intervention^c^	5.7 (6.2)	5.6 (5.3)	*—*

a *Mean change in count of patients adjusted using full pre-specified model, including baseline mean number of patients with PIP, proportion of patients living in deprivation, and GP practice locality.*

b *Single agent or in combination.*

c *Mean count of patients with PIP. CI = confidence interval. ICS = inhaled corticosteroids. LABA = long-acting β-agonist. LAMA = long-acting muscarinic antagonist. PIP = potentially inappropriate prescribing. SABA = short-acting β-agonist. SD = standard deviation.*

### Process evaluation findings

In total, 208 practices (88.5% return rate) returned at least one survey, and 70 (29.8%) practices returned two surveys. There was no difference in response rate between intervention (90.7%) and control (87.8%) practices (Supplementary Table S5). If there were two returns, the survey evaluated was that which had been received from a member of the practice team rather than the prescribing support team.

A majority of intervention practices found the background information and patient-level data to be moderately or very useful (72.9% and 83.2%, respectively), and the majority of practices (69.2%) reported that the feedback was discussed among the practice team on multiple occasions (Supplementary Table S5).

Most intervention practices reviewed some or all patient records (80.4%), flagged some or all patient records (77.6%), or consulted with some or all patients face-to-face (62.6%) (Supplementary Table S5). In intervention practices that reported this (*n* = 18), the removal of SABA inhalers from repeat to acute prescribing (thereby increasing clinical oversight) was the most common change to prescription processes (Supplementary Table S6).

## DISCUSSION

### Summary

This pragmatic cluster RCT found that practice feedback on PIP of asthma bronchodilator inhalers that included practice-specific background information, patient-level visualised medication histories, and action-oriented messages resulted in a statistically significant reduction in the number of patients who experienced PIP of bronchodilator inhalers. There were statistically significant reductions in four of the five individual measures that made up the composite measure of PIP of bronchodilator inhalers. In the process evaluation, the majority of practices reported that they found the feedback content useful and reported discussing the feedback in the practice.

### Strengths and limitations

The main strengths of this study are that it was conducted to high standards for RCTs, that the intervention was designed to include features identified as effective by a Cochrane review of audit and feedback,^10^ that it was pragmatic^15^ (being run in routine practice, with feedback using administrative data), and that all eligible practices in the health board were sent feedback.^16^

Limitations included the fact that researchers were not blind to the intervention allocation as they worked in the clinical service — although it should be noted that randomisation was independent and blinded, and analysis was blinded. In addition, there was only one active follow-up with practices during the intervention period, although this reflected the resource constraints of everyday improvement practice. The feedback data did not include diagnostic information, so some of the people identified may have had non-asthma reasons for being prescribed bronchodilators; however, measures were designed to excluded people likely to have COPD based on prescribing patterns.^17^

It is also possible that feedback resulted in unintended harm by inhibiting appropriate bronchodilator inhaler prescribing: changes to prescribing processes may have made it harder for bronchodilator inhalers to be re-ordered or, because feedback focused attention on asthma prescribing, it may have overshadowed the paying of attention to other conditions.^18^ Resource constraints in this pragmatic trial meant that such potential adverse effects could not be evaluated, but both of these risks are routine in almost all health service prescribing quality improvement activity.

Resource constraints also meant that the process evaluation had no open questions, although the authors recognised that these may have yielded useful feedback from practices in terms of how to refine the intervention and to what extent further improvement might be achieved.

Another limitation of the study was that outcomes were only measured using prescription data. It was not possible to link to other data sources and, thereby, determine how the changes in prescribing might have affected the use of other NHS services.

### Comparison with existing literature

Audit and feedback is a widely used intervention to improve professional practice.^10^

Simple feedback of the number of patients with excess bronchodilator inhaler prescribing in asthma alone^19^^,^^20^ or within an educational intervention^21^^–^^23^ has been shown to have no discernible impact on bronchodilator inhaler prescribing; in none of these studies — unlike the one presented here — were patient details or medication histories for individual patients provided.

Complex interventions in other therapeutic areas where feedback on prescription data was provided with other components additional to education (for example, quality-improvement methodology, financial incentives, integration of feedback in live health records, and behavioural-change messaging) showed improvements in high-risk prescribing^24^^–^^26^ and treatment costs.^27^

### Implications for research and practice

This low-intensity feedback intervention is pragmatic and sustainable because it uses administrative prescription data, which provides consistent and accurate information that is regularly updated. Although the effect size is relatively small, feedback has the advantage of being simpler and cheaper to deliver compared with educational outreach, pay-for-performance, or performance target-setting and management systems. The study presented here provides some evidence that detailed and well-designed feedback, with no other additional intervention components, can be sufficient to improve prescribing behaviour. Linking to diagnosis would improve the measures relating to the PIP of bronchodilator inhalers, ensuring feedback focuses specifically on asthma and excludes those patients with COPD.

Published research indicates that PIP for asthma occurs in other countries^19^^,^^20^^,^^22^^,^^23^^,^^28^ and, therefore, this intervention might be generalisable beyond the NHS and the UK; a similar intervention could be developed and implemented in any country or health system in which prescription data are consistent, comprehensive, longitudinal, patient identifiable, and can be attributed to practices or individual prescribers.

Important areas for future research include: understanding whether low-intensity interventions such as the one reported here have a sustained effect, with or without ongoing feedback; for which therapeutic topics it will be most effective; how it can be enhanced to improve its effectiveness; and when other types of interventions would be better suited. Qualitative methods could be used to better understand practitioner perspectives.

This study found that a pragmatic patient-level data feedback intervention embedded in routine prescribing improvement activity led to a statistically significant improvement in the number of patients exposed to excess or unsafe prescribing of bronchodilator inhalers. The intervention would be feasible to implement at scale in contexts that have electronic prescribing in general use.

## Data

The data controller of the data analysed is NHS National Services Scotland. Patient-level data are available subject to standard information-governance requirements for the use of anonymised, unconsented NHS Scotland data (http://www.informationgovernance.scot.nhs.uk/pbpphsc/home/for-applicants).
